# Baseline estimation of cognition with retinal thickness in aging and Alzheimer disease: an ONDRI study

**DOI:** 10.1002/alz70861_108875

**Published:** 2025-12-23

**Authors:** M. Amin Banihashemi, Faryan Tayyari, Morgan Koo, Malcolm Binns, Paula M McLaughlin, Donna Kwan, Mario Masellis, Richard H. Swartz, Peter J. Kertes, Wendy Hatch, Christopher Hudson, Maged Goubran, Sandra E. Black, ONDRI Investigators

**Affiliations:** ^1^ Institute of Medical Sciences, The Temerty Faculty of Medicine, University of Toronto, Toronto, ON Canada; ^2^ School of Optometry and Vision Science, University of Waterloo, Waterloo, ON Canada; ^3^ Sunnybrook Research Institute, Toronto, ON Canada; ^4^ Dalla Lana School of Public Health, University of Toronto, Toronto, ON Canada; ^5^ Baycrest Academy for Research and Education, Toronto, ON Canada; ^6^ Nova Scotia Health, Halifax, NS Canada; ^7^ Queens University, Kingston, ON Canada; ^8^ Cognitive and Movement Disorders Clinic, Sunnybrook Health Sciences Center, Toronto, ON Canada; ^9^ Division of Neurology, Department of Medicine, Sunnybrook Health Sciences Centre, Toronto, ON Canada; ^10^ The John and Liz Tory Eye Centre, Toronto, ON Canada; ^11^ Department of Ophthalmology and Vision Sciences, University of Toronto, Toronto, ON Canada; ^12^ Artificial Intelligence and Computational Neurosciences Lab, Sunnybrook Research Institute, University of Toronto, Toronto, ON Canada; ^13^ Department of Medical Biophysics, University of Toronto, Toronto, ON Canada; ^14^ Dr. Sandra E. Black Centre for Brain Resilience and Recovery, LC Campbell Cognitive Neurology, Hurvitz Brain Sciences Program, Sunnybrook Research Institute, University of Toronto, Toronto, ON Canada; ^15^ Ontario Neurodegenerative Disease Research Initiative, Toronto, ON Canada

## Abstract

**Background:**

The contribution of retinal total thickness in optical coherence tomography (OCT) imaging towards estimating a comprehensive cognitive profile in aging and dementia is not well studied. To investigate the contribution of retinal total thickness towards comprehensively estimating cognitive functions.

**Methods:**

This was an observational cross‐sectional study. Participants were from the Ontario Neurodegenerative Research Initiative (ONDRI) study from Ontario, Canada. One hundred thirty‐three adults across aging‐mild cognitive impairment (MCI)‐Alzheimer disease (AD) were included. Outcomes were scores across attention & working memory, language, executive, memory, and visuospatial domains and Montreal Cognitive Assessment total score. Measures included macular and peripapillary retinal nerve fibre layer total thickness measured by retinal OCT. Analysis considered age, sex, years of education, and diagnosis. Cognitive measures were estimated using ordinary least squares (OLS) and partial least squares (PLS) regression.

**Results:**

Mean age was 70±8 and 56% (75/133) were women. Diagnostic groups included normal aging (*n* =44), MCI (*n* =58), and AD (*n* =31). Macular volume/thickness, particularly non‐foveal thickness, estimated language composite scores in all participants via OLS (R^2^ = 0.24–0.29, β = 0.17–0.25, SE = 0.08, *p* < 0.05). The contribution of retinal thickness towards estimating cognitive measures was markedly improved in the AD subgroup using PLS to estimate MoCA total score and composite scores of executive functions, and attention and working memory (f^2^ = 0.04–0.13, SE = 0.01–0.02). Retinal thickness also contributed towards estimating attention and working memory, and language in normal aging (f^2^ = 0.02–0.04, SE = 0.00–0.01). There seemed to be greater estimation of ventral visual stream functions (e.g., object recognition) or switching tasks. However, the overall explanatory ability of both OLS and PLS models was low (R^2^ < 60%) with many non‐significant associations in OLS. Adding retinal thickness actually slightly lowered the explanatory ability of 73–91% of OLS and PLS models respectively in all participants (f^2^<0).

**Conclusions:**

Retinal thickness may estimate functions related to attention and executive function in later disease stages, but language appears to be better estimated in normal controls.

**Additional information**: Canadian Institutes of Health Research fund reference number: 177881.

